# Synthesis of acrylic resin and methacrylic resin microspheres by suspension polymerization

**DOI:** 10.3389/fchem.2023.1193553

**Published:** 2023-06-09

**Authors:** Han Yu, Junjie Liu, Lin Zhao, Yonglin Liu, Lulu Gu, Lianxiang Feng, Yonggen Weng, Qingxu Duan, Baorong Duan, Jiale Qu

**Affiliations:** ^1^ Research Center for Leather and Protein of College of Chemistry and Chemical Engineering, Yantai University, Yantai, China; ^2^ Institute of Rehabilitation Engineering, Binzhou Medical University, Yantai, China; ^3^ Department of Physics, Binzhou Medical College, Yantai, China; ^4^ School of Environmental and Municipal Engineering, Qingdao University of Technology, Qingdao, China; ^5^ Tancheng County branch of Linyi Ecological Environment Bureau, Linyi, China; ^6^ Qihe Leahou Chemical Co., Ltd., Dezhou, China

**Keywords:** suspension polymerization, polymer microspheres, methyl methacrylate, methyl acrylate, acrylate resin

## Abstract

The process of suspension polymerization was utilized to create acrylate resin microspheres with mesh numbers of 140–200 μm and particle sizes of 100 μm for implementation in mesh coating technology. The copolymer of methyl methacrylate (MMA) and methyl acrylate (MA) served as the primary polymer, with dibenzoyl peroxide (DBPO) functioning as the initiator, and a mixture of calcium carbonate and deionized water served as the dispersion medium. The surface morphology of the synthesized microspheres was analyzed through Fourier-transform infrared (FTIR) spectroscopy and scanning electron microscopy (SEM) to confirm successful synthesis. The optimal reaction conditions for the synthesis of these microspheres were determined to be a dispersant dosage of 30 g of calcium carbonate with a monomer ratio of 4:1, a reaction time of 1 h, an initiator dosage of 1.2 g of BPO, and a reaction temperature of approximately 75–80 C, resulting in microspheres with a regular spherical shape and smooth surface.

## 1 Introduction

Suspension polymerization is a ubiquitous and effective method for the synthesis of polymer microspheres (Kim et al., 2012; Kang et al., 2015; Lobry et al., 2015; Norhayati et al., 2018; Ali et al., 2019; Kang et al., 2019; Denisha Jayaweera and Narayana, 2021; Moratille et al., 2022; Park et al., 2022; Vu Nguyen et al., 2023). This approach has been widely used in the production of acrylate polymer microsphere coatings, which possess several advantages, including energy efficiency, resource conservation, and environmental compatibility (Song et al., 2019; Zhong et al., 2021). The acrylate resin and the corresponding curing agent are the primary components of this variety of coating, which is known for its excellent decorative and weathering properties. The acrylate resin coating demonstrates impressive resistance to aging, corrosion, abrasion, and outdoor exposure, and boasts excellent color retention and high mechanical properties, as well as flexibility and hardness. These qualities make it an ideal choice for use in a variety of industries, including household appliances, decoration, and beauty products (Wang et al., 2000). Compared to other types of coatings, such as epoxy and polyester, acrylate resin coating is a high-grade material that is rapidly being developed and improved.

In a study conducted by Zhang et al., monomers such as kappa-butyrolactone and vinyl acetate were used with sodium acetate and polyvinyl alcohol serving as dispersants, and high-pressure synthesis was applied to produce spherical acrylate microbeads (Liu et al., 2020). The spherical microbeads were synthesized with a large diameter, and they failed to meet the size requirements for microbeads in cosmetic products. In order to ensure the suitability of microspheres for use as a human nail coating, the particle size must neither be very large nor very small. If the particle size is very small, the fluidity is very large, making it difficult to control. If the particle size is very large, the application’s flatness is very low and the customer’s skin sensation is poor (Chen and Gao, 2002; Ballard et al., 2017; Yuan et al., 2017; Liu et al., 2019; Shin Kim et al., 2021). Considering the customer’s overall requirements, the particle size distribution should be 75–110 μm (during the process of filtering fine particles, the mesh specifications utilized ranged from 140 to 200 mesh), which can meet the customer’s requirements (Liu et al., 2000; Chen and Gao, 2002; Qin and Yang, 2004; Liu and Wang, 2010; Chen and Zhang, 2019).

The present experiment uses suspension polymerization to synthesize acrylic resin microspheres with a yield of 91.73%. The particle size of the resulting microspheres is uniform and smooth with a yield of 140–200 mesh as high as 61.47%. The color of the microspheres is processed through a specialized process, resulting in a new type of nail polish. This research utilizes methyl methacrylate (MMA) and methyl acrylate (MA) as raw materials, along with benzoyl peroxide as the initiator, to synthesize a uniform, smooth-surfaced polymer. The production of acrylate resin microsphere coating represents a significant advancement in the field of polymer synthesis, offering a high-quality, efficient, and widely used solution for a variety of industrial and consumer applications.

## 2 Results and discussion

### 2.1 Screening dispersant species

The utilization of magnesium carbonate (MgCO_3_) and calcium carbonate (CaCO_3_) as dispersants yielded disparate results in the synthesis of the polymer (Table 1). MgCO_3_ imparted a gray hue to the final product, while CaCO_3_ produced a more brilliant, white polymer. In light of the specific requirements of the product, the experiment used CaCO_3_ as the dispersant. The choice of dispersant played a key role in determining the final color and appearance of the polymer, highlighting the importance of carefully selecting appropriate materials for a given application.

**TABLE 1:** Effect of dispersant species on the product.

Dispersant species	Product status
MgCO_3_	Gray and lower yield
CaCO_3_	White, bright, and high yield

### 2.2 Effect of dispersant dosage on the product

As evidenced in Table 2, the actual amount of the dispersant employed exerted a significant influence on the uniformity, particle size, and yield of the resulting particles. It has been observed that when the organic dispersant is completely dissolved, the relative molecular weight of the polymer gradually increases with the continued progression of the polymerization reaction. Furthermore, a concentration of the dispersant that is sufficient to form an effective protective film on the surface of the droplet can aid in the timely prevention and reduction of droplet agglomeration. These factors collectively impact the overall properties of the resulting particles.

**TABLE 2:** Effect of dispersant dosage on the product.

CaCO_3_/g	Yield/g	Productivity/%	Mesh number (50)/%	Mesh number (100)/%	Mesh number (140)/%	Mesh number (200)/%
20	23.49	78.30	3.13	0.95	0.62	0.32
25	24.56	81.87	80.3	12.4	4.5	1.2
30	27.52	91.73	17.56	8.62	25.61	35.86
35	19.73	65.77	47.13	15.71	11.28	11.71
40	18.34	61.13	11.6	10.54	13.49	46.57

As the concentration of CaCO_3_ increased, the particle size of the polymer beads decreased dramatically. This reduction in particle size was primarily attributed to the increased wrapping of CaCO_3_ particles in the outer layer of monomer droplets, which hindered the uniform cohesion between the droplets, thereby ensuring the stability of the suspension system and the dispersion. However, it should be noted that an excessively high concentration of the dispersant can lead to an increase in the number of nucleation particles, resulting in a decrease in the amount of monomers obtained from each particle and ultimately leading to a smaller particle size. Conversely, a low dispersant concentration may result in weak dispersion and an increase in monomer viscosity, leading to agglomeration. When the dispersant concentration was insufficient, the stability of the polymerization system was impaired, and cohesion phenomena occurred in the polymer particles. When the dispersant concentration was very high, the polymer beads were found to be smaller in size. The results indicated that the optimal dosage of the dispersant for CaCO_3_ was 30 g, with polymer beads having a particle size of 140 to 200 mesh, accounting for the highest proportion.

### 2.3 Effect of the monomer ratio on the product

As outlined in Table 3, the monomer ratio has a profound impact on the degree of caking, color, and yield of the reaction product. In this particular case, the proportion of MMA was relatively low, while the proportion of MA was relatively high. This configuration of monomers created a system with a significant degree of cohesion, making it challenging to achieve adequate stirring, mass transfer, and heat transfer, ultimately leading to an out-of-control polymerization reaction, system caking, and the formation of a yellowish color. Conversely, as the proportion of MMA increases, the reaction product aggregates become smaller and the yield increases. Furthermore, when the ratio of MMA to MA is 4:1, the yield of the target polymer product (mesh number ranging from 140 to 200) was found to be exceptionally high, thereby fulfilling the majority of the polymer and composite process requirements. When the ratio of MMA to MA is 5:1, the yields of the target polymer product and the total polymerization product (Productivity) are both found to be decreased, indicating that the chemical reaction between individual molecules is not sufficient to achieve optimal results. Therefore, when the ratio of MMA to MA is 4:1, the reaction effect is found to be most optimal.

**TABLE 3:** Effect of the monomer ratio on the product.

MMA/g	MA/g	Yield/g	Productivity/%	Color	Mesh number (140)/%	Mesh number (200)/%
6	24	Caking	0.00	Cream yellow	0	0
15	15	Caking	0.00	Cream yellow	0	0
18	12	15.59	51.97	Cream yellow	0.95	0.38
21	9	18.10	60.33	Cream yellow	4.70	2.10
24	6	27.52	91.73	White	22.19	25.15
25	5	14.71	49.00	White	1.09	0.14

### 2.4 Effect of the initiator type on the product

According to Table 4, it can be seen that at a same initiator amount, the BPO and azobisisobutyronitrile (AIBN) initiators differ in their efficacy in the synthesis of macroscopic particles. When the synthesized particles meet the process requirements under the action of the AIBN initiator, the amount of coagulants produced is significantly reduced and the proportion of coagulants that meet the requirements of the copolymerization process (140–200 mesh) is relatively low. On the other hand, under the action of the BPO initiator, the amount of coagulants produced is much larger and the proportion of coagulants that meets the requirements of the copolymerization process is much higher. Furthermore, the efficacy of the initiator is also improved when the amount of the BPO initiator is increased from 0.6 g to 1.2 g. These results suggest that the BPO initiator is more effective than the AIBN initiator in the synthesis of macroscopic particles that meet the requirements of the copolymerization process.

**TABLE 4:** Effect of the initiator type on the product.

Type	Quantity/g	Yield/g	Productivity/%	Mesh number (50)/%	Mesh number (100)/%	Mesh number (140)/%	Mesh number (200)/%
AIBN	0.6	22.71	75.70	50.7	18.9	5.3	1.8
AIBN	1.2	23.09	76.97	32.1	24.0	13.5	16.5
BPO	0.6	24.20	80.67	43.8	19.7	12.9	2.4
BPO	1.2	27.52	91.73	17.56	8.62	25.61	35.86

### 2.5 Effect of initiator dosage on the product

It can be seen in Table 5 that with the increase of the initiator dosages, the yield changes a little; however, it had a greater impact on the particle size. The reason was with the increase in the number of initiators, the concentration of free radicals that can participate in the reaction also increases and the reaction rate increases so that the polymerization center of the monomer proliferates, which was conducive to the formation of numerous and smaller diameter particles. Although when the quantity of the initiator is scarce, the number of polymerization centers that can be formed is limited. Under these conditions, the reactant molecules ultimately form larger particles with a diameter centrally located due to the relatively few polymerization centers. Therefore, with the increase in the amount of the initiator (BPO), the proportion of polymer products gradually decreased under 50 mesh and the proportion of polymer products gradually increased under 140 mesh and 200 mesh, which produces the product we desired. Nevertheless, too many initiators are also unnecessary and will not lead to better outcomes. When the number of initiator exceeds a certain threshold, the resulting polymer particles have a diameter lower than the target product’s standard (mesh number >200). Therefore, when the initiator (BPO) was 1.2 g, the proportion of polymers that meets the process requirements was the highest and the effect was the best.

**TABLE 5:** Effect of initiator dosage on the product.

Initiator quality/g	Yield/g	Productivity/%	Mesh number (50)/%	Mesh number (100)/%	Mesh number (140)/%	Mesh number (200)/%
0.3	25.3	84.33	46.26	16.12	9.87	2.31
0.6	24.2	80.67	43.5	21.7	12.9	2.4
0.9	26.6	88.67	34.9	26.3	21.1	7
1.2	27.52	91.73	17.56	8.62	25.61	35.86

### 2.6 Effect of temperature on products

As demonstrated in Table 6, the influence of temperature on various characteristics of the particles was quite inconsistent. When the degree of uniformity, transparency, and productivity were relatively unaffected by temperature changes, the degree of cohesion, particle size, and the time of concentrated polymerization were found to be more sensitive to temperature fluctuations. Specifically, it was observed that as the temperature increased, the time required for the polymerization reaction decreased, albeit with the potential for accumulation and explosion when the temperature became very high. Conversely, when the temperature was lowered, polymerization time increased, although the optimal temperature range for comprehensive consideration of temperature effects was found to be approximately 75–80°C, where the degree of cohesion, size, and time of concentrated polymerization could be balanced.

**TABLE 6:** Effect of temperature on the product.

T/°c	Product appearance	Caking degree	Transparency	Productivity/%		Aggregation time (h)
70–75	Uniform particle	Small	Great	74.67		2.5
75–80	Uniform particle	None	Great	71.21	1.5 h	
80–85	Uniform particle	Big	Great	70.51	Caking	

### 2.7 Time of reaction

As shown in Table 7, the results showed that the proportion of polymers under each mesh number shows a little change with the advancement of time. From an energy-saving perspective, it was found that the reaction time of 1 h was the most cost-effective.

**TABLE 7:** Effect of reaction time on the product.

Time/h	Yield/g	Productivity/%	Mesh number (50)/%	Mesh number (100)/%	Mesh number (140)/%	Mesh number (200)/%
1	16.88	56.27	34.72	22.61	16.61	10.60
2	14.62	48.74	40.16	28.01	18.52	8.27
3	14.63	48.77	40.63	23.64	16.44	9.20
4	14.60	48.69	41.52	25.86	14.51	7.77
5	10.86	36.20	38.72	20.27	12.14	7.40

However, when the reaction time between the suspension and the polymerized material was excessively short, the beads in the suspension polymer reaction were not entirely hardened, making it easier for the beads of different polymer materials to stick to each other, resulting in an inaccessible product with a disordered bead shape. Conversely, an extended duration of polymerization had little impact on the change in the particle size of all polymerization products, indicating that after the polymerization duration reached 1 hour, the monomers had transformed from solid droplets to solid beads of the copolymer. The hardening of the dots was complete, the reaction duration was gradually extended, and the particle size no longer changed. Furthermore, prolonging the polymerization reaction time did not result in an increase in yield.

### 2.8 Infrared spectrum analysis

**FIGURE 1:**
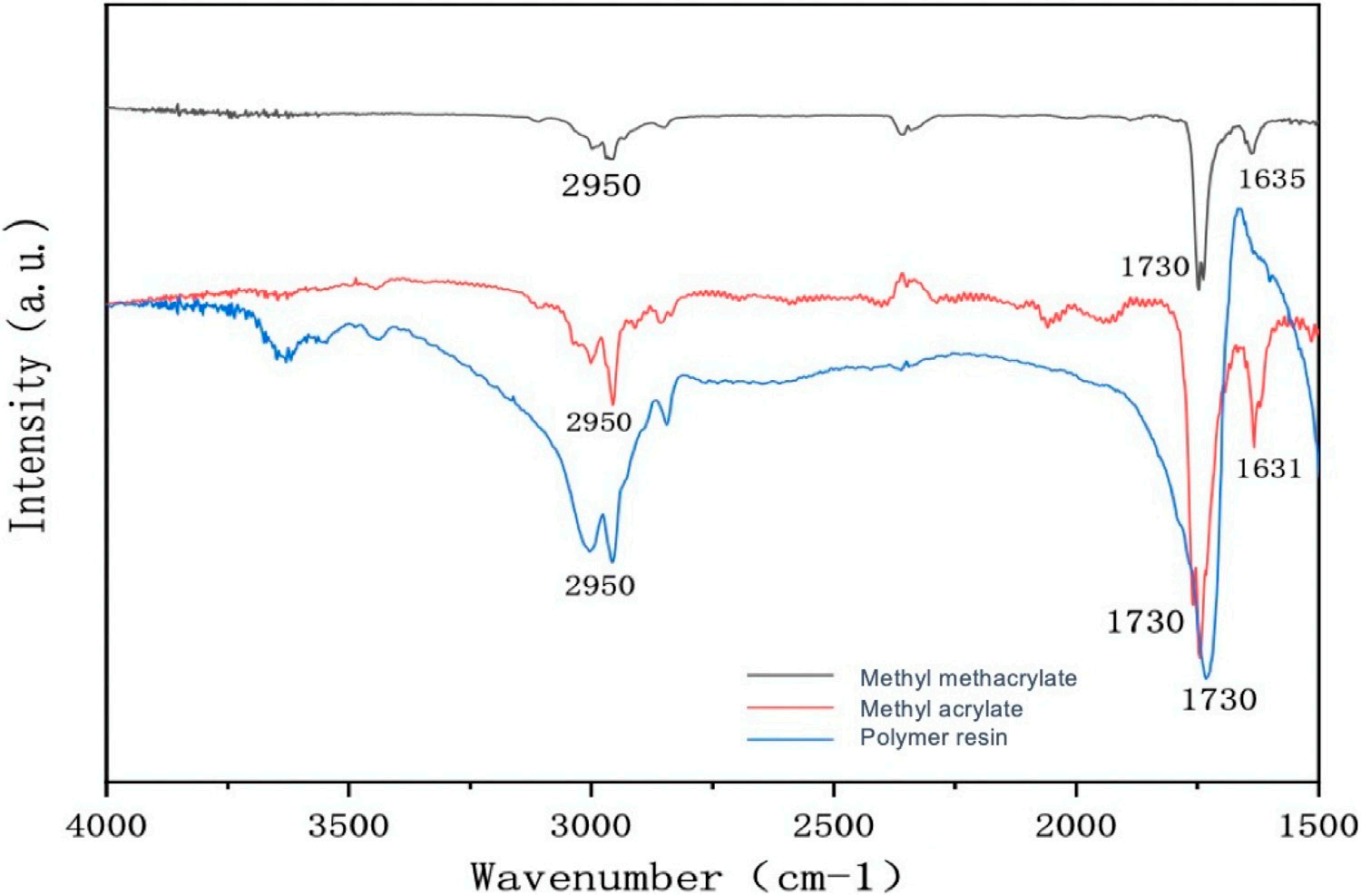
Infrared spectrum of methyl methacrylate (black line), methyl acrylate (red line), and polymer resin (blue line).

As shown in Figure 1, the sample was subjected to infrared characterization, following its dissolution in potassium bromide and compression into tablets. The absorption peak at approximately 2,950 cm^−1^ is attributable to the stretching vibration of the methyl (-CH_3_) group, while the peak at around 1,730 cm^−1^ represents the carbonyl group (C=O) stretching vibration. The presence of these peaks suggests that the sample contains MMA and MA, which are characterized by their carbon–carbon double bonds (C=C) stretching vibration peaks in the range of 1,631–1,635 cm^−1^. However, it is noteworthy that the absence of stretching vibration peaks in the range of 1,631–1,635 cm^−1^ in the polymer resin indicates that MMA and MA have undergone polymerization to form acrylate resin microspheres without double bonds.

### 2.9 Analysis of polymer micromorphology and relative molecular mass

**FIGURE 2:**
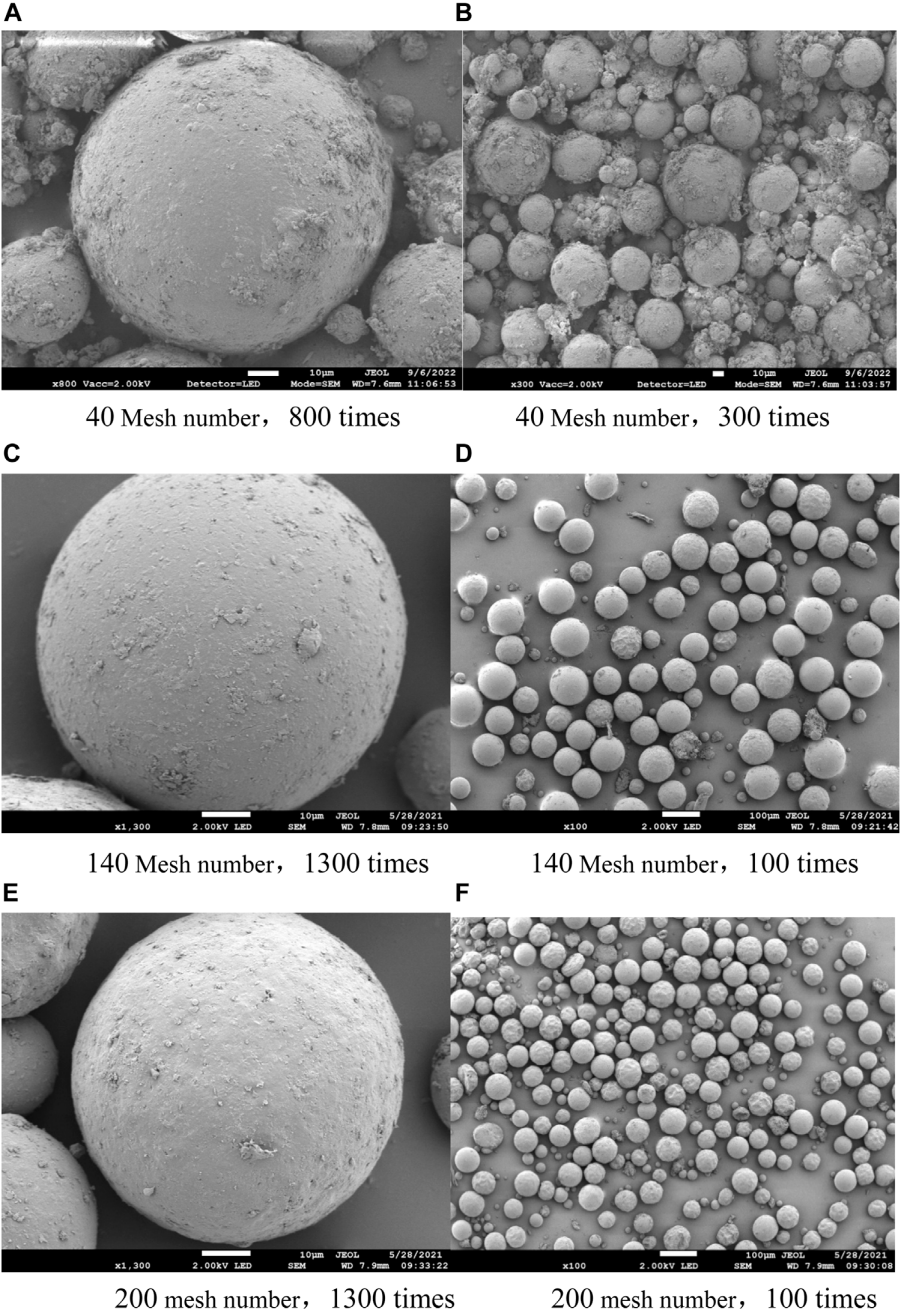
Scanning electron microscope (SEM) of polymer microspheres. **(A)** 40 mesh number, 800 times; **(B)** 40 mesh number, 300 times; **(C)**140 mesh number, 1300 times; **(D)** 140 mesh number, 100 times; **(E)** 200 mesh number, 1300 times; **(F)** 200 mesh number, 100 times.

The utilization of polymer microspheres in nail polish is primarily directed toward enhancing the tactile properties of the formulation. As shown in Figure 2, the prepared microspheres were found to be uniformly spherical in shape and possessed smooth surfaces, with an average particle size of approximately 100 μm. This morphology is crucial for ensuring the desired rheological properties of the final product and for achieving the desired sensory characteristics. The regular and stable nature of the microspheres also confirms their suitability for use in nail polish formulations. Overall, the results indicate that the prepared microspheres are well-suited for use in nail polish applications, offering a significant improvement in terms of the product’s feel and texture.

Employing gel permeation chromatography (GPC) characterized the relative molecular weight of the aggregates obtained under optimal conditions; the results of the test are shown in Table 8 and Supplementary Figures S2, S3, where the average molecular weight is 61,579 and the weight average molecular weight is 277,767. According to the molecular weight formula provided in Supplementary Material 1.6.5, it can be inferred that if the dispersity of the polymer chain is large, the difference between Mn and Mw will be more significant. We can also observe from the diversity of particle sizes of the synthesized polymer that we have obtained a highly branched polymer. Thus, the significant difference in the molecular weight of the obtained aggregates suggests that there is room for improvement in the synthesis method and reaction speed to control the molecular weight of the aggregates.

**TABLE 8:** Relative molecular mass of the polymer (g/mol).

Mp	Mn	Mw	Mz	Mz+1
185,440	61,579	277,767	719,741	1,376,869

### 2.10 Polymer microsphere skin feel test

As demonstrated in Table 9, the incorporation of polymer microspheres into the experimental product was found to result in improved smoothness and application properties. The spherical shape of the polymer particles facilitated a rolling effect during application, making it easier for the applicator to spread the product evenly. Furthermore, the microspheres were found to provide a smooth, velvety feeling upon application, as opposed by the products without the addition of microspheres, which tended to leave a less soft feeling after use. To gain further insight, 10 volunteers were recruited to test the products and provide their feedbacks (the full score is 5). The results revealed that the volunteers greatly appreciated the nail polish formulations prepared through suspension polymerization, particularly those containing microspheres, which were found to be less irritating than the commercial products. However, the formulations with microspheres were noted to have a greasy texture and the powder was prone to uneven distribution, indicating the need for further optimization. Nevertheless, the feedback from volunteers was highly favorable, with attributes such as long retention time, simple operation steps, fast-drying mechanism, a non-essential nail phototherapy lamp, time-saving attribute, high color rendering, and unwarranted repeated coloring, among the characteristics highly appreciated by the testers.

**TABLE 9:** Effects of experimental articles and samples on skin feeling (a score of 5 represents the highest level of satisfaction).

	Smoothness on application	Ductility	Thrill	Greasy feeling	Skin sensation
Experimental products	4.3	4.3	3.6	4.4	4.3
Commercial samples	4.0	3.9	4.0	4.1	4.1

## 3 Conclusion

The present study aimed to synthesize polymer microspheres of varying particle sizes through suspension polymerization. The impact of diverse preparation conditions on the stability of the polymerization reaction and the size of the polymer microspheres were investigated. Additionally, the properties and microstructures of the microspheres were characterized. The research focused on the application properties of polymer microspheres after the preparation of nail polish. In particular, the research study aimed to determine the optimal reaction conditions for the synthesis of nail polish using MMA and MA via the suspension polymerization method. CaCO_3_ was utilized as a dispersant, with a dosage of 30 g, and the monomer ratio was set at 4:1. The reaction time was fixed at 1 h, and the initiator used was BPO, with a dosage of 1.2 g. The reaction was carried out at a temperature range of 75–80°C. The polymer microspheres obtained from the reaction exhibited a regular spherical shape and smooth surface. The study also examined the application properties of polymer microspheres after the preparation of nail polish. The nail polish prepared from the polymer microspheres displayed excellent spreadability and smoothness, making it highly acceptable to the skin.

## Data Availability

The original contributions presented in the study are included in the article/Supplementary Material. Further inquiries can be directed to the corresponding authors.
