# Supplementary material for: Synthesis of acrylic resin and methacrylic resin microspheres by suspension polymerization
Source: Front Chem. 2023 Jun 9;11:1193553. doi: 10.3389/fchem.2023.1193553 (PMC10288845; doi:10.3389/fchem.2023.1193553)
Supplement: Supplementary file 1 [file Presentation1.pdf]

Supporting Information for "Synthesis of Acrylic Resin and Methacrylic Resin Microspheres by Suspension Polymerization

Han Yu[2,1, §] · Junjie Liu[3,§] ·Lin Zhao[1] · Yonglin Liu[4] · LuLu Gu[5] · Lianxiang Feng[6] · Yonggen Weng[1] · Qingxu Duan[1] · Baorong Duan[1,*] · Jiale Qu[2,*]

1. Research Center for Leather and Protein of College of Chemistry & Chemical Engineering , Yantai University, Yantai 264005, China

2. Institute of Rehabilitation Engineering, Binzhou Medical University, Yantai 264003, China

3. Department of Physics, Binzhou Medical Ccollege, Yantai 264003, China

4. School of Environmental and Municipal Engineering, Qingdao University of Technology, Qingdao 266525, China

5. Tancheng County branch of Linyi Ecological Environment Bureau, Linyi 276100, China

6. Qihe Leahou Chem COLTD, Dezhou 251199, China

§ Author contributions: These authors contributed equally to this work.

*corresponding author: qujiale@buaa.edu.cn; duanbaorong@126.com

1 Materials and Methods

1.1 Experimental principle

Acrylate resin is synthesized from methyl methacrylate and methyl acrylate by suspension polymerization. The reaction formula is as follows:

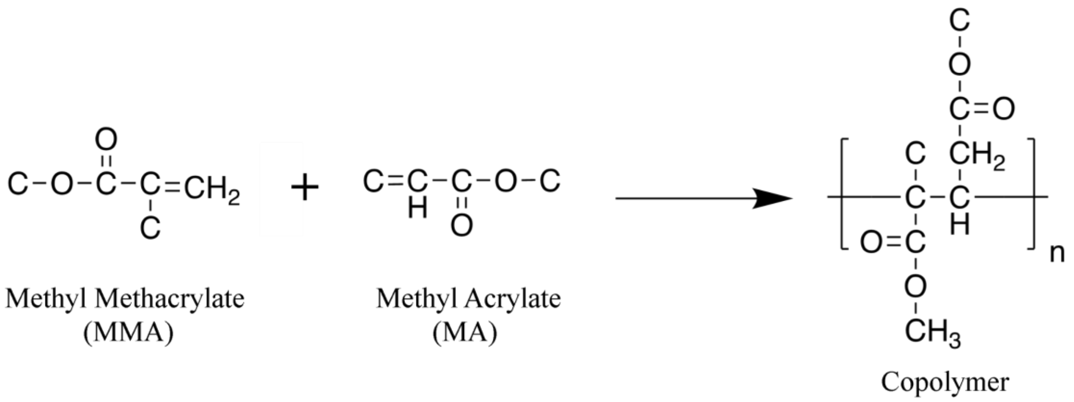

Figure S1. Suspension polymerization product synthesis equation

1.2 Chemicals and instruments

Methyl methacrylate (analytical grade) was purchased from the Tianjin Zhiyuan Chemical Reagent Co., Ltd.; Methyl acrylate (analytical grade) was purchased from the Tianjin Damao Chemical Reagent Factory; Calcium carbonate (analytical grade) was purchased from the Tianjin Beichen Fangzheng Reagent Factory; Magnesium carbonate (analytical grade) was purchased from the Tianjin Beichen Fangzheng Reagent Factory; Benzoyl peroxide (analytically pure) was purchased from the Tianjin Beilian Fine Chemicals Development Co., Ltd.,; Azobisisobutyronitrile (analytically pure) was purchased from the Tianjin Beilian Fine Chemicals Development Co., Ltd.; Ten Sodium dialkylbenzene sulfonate (chemically pure) was purchased from the Tianjin Bodi Chemical Co., Ltd.; Hydrochloric acid (analytically pure) was purchased from the Yantai Sanhe Chemical Reagent Co., Ltd.

Electric heating constant temperature blast drying oven, DHG-9073, Shanghai Youyi Instrument Co., Ltd.; Oil bath, HH-DR1, Shanghai Chengjie Instrument Equipment Co., Ltd.; Electronic balance, JJ22ABC, Changshu Shuangjie Testing

Instrument Factory; Vacuum pump, SH_2-D, Gongyi Yuhua Instrument Co., Ltd.; Scanning Electron Microscope, JSM-7900F, Nippon Electronics Corporation; Sieve, GB/T6003.1-2012, Shangyu Hujiang Instrument, Shaoxing City.

1.3 Experimental method

Step 1: Blending MMA and MA, followed by the addition of BPO and thorough mixing.

Step 2: First, in three beakers, add $CaCO_3$ and deionized water, and mix while heating to 60°C, allowing $CaCO_3$ to fully dissolve. Next, add the pre-processed MMA and MA mixture, and with deionized water rinse the MMA and MA flask, and add the entire contents to the three beakers. Heat the mixture to 75°C, adjust the mixing speed, and maintain a constant mixing speed for approximately 1 hour. Then, withdraw a small amount of bead-like precipitate using a dropping tube, and allow it to cool in water. If it does not harden, continue the reaction while adjusting the mixing speed to slow down the reaction, allowing the single species to fully react. If it hardens, increase the temperature to speed up the hardening process, and heat the mixture to 80°C, reacting at a constant speed for 3 hours.

Step 3: Transfer the product to a large flask, cool, and allow the supernatant to be decanted. Add HCl (10%) and mix, adjusting the pH to 1-1.5.

Step 4: Separation and drying of the polymer: Repeat the addition of cold deionized water three times to wash the precipitated polymer, and then filter. Dry the filtered polymer in an oven at a temperature of 70°C for 6 hours.

1.4 Modulate the reaction parameters to investigate their influence on the product formation

In this experimental study, an exhaustive examination of the reaction conditions was undertaken by manipulating parameters such as temperature, time, the calcium carbonate concentration, the molar ratio of MMA to MA, the type and concentration of initiator, and the amount of initiator [1].

1.5 Initiator half-life and dosage

$$\text{BPO} \quad t_{\frac{1}{2}} = \frac{0.693}{k_d} = \frac{0.693}{2.5 \times 10^{-5}} = 7.7h$$

$$\text{ABIN} \quad t_{\frac{1}{2}} = \frac{0.693}{k_d} = \frac{0.693}{21.4 \times 10^{-4}} = 1.4h$$

The above-mentioned half-lives of BPO and ABIN meet the required conditions for the initiator of this experiment [2-5].

1.6 Test analysis

1.6.1 Productivity

Weigh and calculate the yield, and observe the shape, color, and transparency of the polymer.

$$Productivity = \frac{\text{Actual polymer mass}}{\text{Monomer quality}} \times 100\%$$

1.6.2 Sieving

Mesh number reflects the particle size, and as the mesh number increases, the particle size decreases. In the characterization of small aggregated particles of a fine

powder, the mesh number is commonly used to represent the size of the particles.

Table S1 Particle size corresponding to the mesh number

Mesh number	25	50	100	140	200
Particle size /μm	700	270	150	110	75

Pour the dried product into a sieve (25 mesh, 50 mesh, 100 mesh, 140 mesh, 200 mesh) and shake it evenly. Weigh the product's weight under each mesh separately, and calculate the proportion.

1.6.3 Fourier transform infrared spectroscopy analysis

The potassium bromide and the sample were formulated into a tablet at a ratio of 200:1, and subsequently introduced into the infrared measurement apparatus, where the computer parameters were adjusted for analysis. By comparing the chemical bonds of the molecules, corresponding to the characteristic peaks in the infrared spectrum, with the polymer components, the polymer structure was examined.

1.6.4 Analysis of polymer microtopography

The polymer microspheres were subjected to a series of processing steps and subsequently positioned under the electron microscope for visual inspection [6,7].

1.6.5 Molecular weight characterization

We employed the Number-average molecular weight (Mn), weight-average molecular weight (Mw), Z-average molecular weight (Mz), Peak molecular weight (Mp) to characterize the polymer's Relative molecular mass [8,9]. The corresponding molecular weight equations for each type of molecular weight are as follows:

$$M_n = \frac{n_1 M_1 + n_2 M_2 + n_3 M_3 + \cdots}{n_1 + n_2 + n_3 + \cdots} = \frac{\sum n_i M_i}{\sum n_i}$$

$$M_w = \frac{w_1M_1 + w_2M_2 + w_3M_3 + \cdots}{w_1 + w_2 + w_3 + \cdots} = \frac{\sum w_iM_i}{\sum w_i}$$

since $n_i = \frac{w_i}{M_i}$, so

$$M_w = \frac{w_1M_1 + w_2M_2 + w_3M_3 + \cdots}{w_1 + w_2 + w_3 + \cdots} = \frac{\sum w_iM_i}{\sum w_i} = \frac{\sum n_iM_i^2}{\sum n_i M_i}$$

$$M_z = \frac{\sum n_iM_i^3}{\sum n_i M_i^2}$$

$$M_{z+1} = \frac{\sum n_iM_i^4}{\sum n_i M_i^3}$$

n_i, the number of moles of polymer with molecular weight Mi; wi, the mass of polymer with molecular weight Mi. Peak molecular weight (Mp) is the molecular weight of the most prevalent type of polymer molecule in the polymer sample and this type of polymer molecule comprise the highest proportion in the total polymer sample.

Under ideal conditions, if the synthesized polymer is composed of identical monomer chains of uniform length (such that all particles are of the same size), then Mn = Mw = Mz = Mz+1 = Mp (Monodisperse polymer). With increasing diversity of monomer chain lengths, the molecular weights characterized by each equationof polymer differ more significantly, and satisfy the rule Mn < Mw < Mz < Mz+1 (Polydisperse polymer) [8,9].

1.6.6 Skin sensation testing

Following the completion of the preparation, the polymer exhibiting optimal reactivity was subjected to dyeing. Eight individuals were conscripted for a blind test to evaluate the efficacy of nails fabricated from the polymer, encompassing aspects such as smoothness after application, spread of application, irritation, greasy sensation,

and overall skin feeling liking degree. The score was calculated as the mean value of all participants, with a lower score indicating a less favorable outcome. Subjectively, a digitized five-point scale was utilized to assess the personal skin sensation, with a lower value corresponding to an increasingly unfavorable sensation.

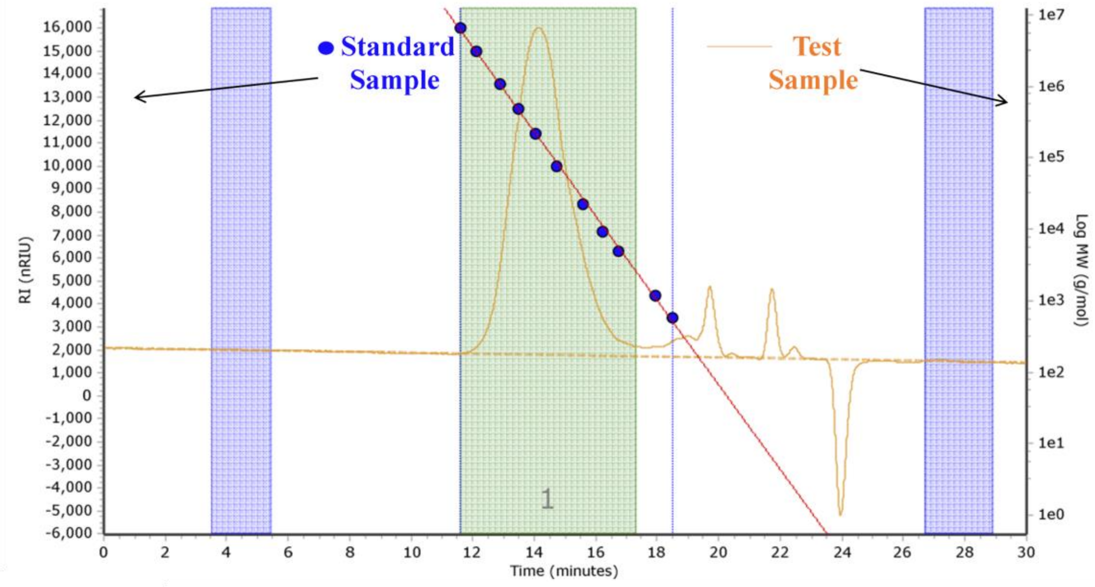

Figure S2. Polymer molecular weight outflow diagram

In Figure S2, the blue dots represent the known molar quantity of the standard sample for the GPS instrument, with the fitted red line and the corresponding Y-axis is on the left side. The curved line depicted in orange represents the test sample, with the corresponding Y-axis on the right side. The x-axis represents time, and the y-axis measures the molecular weight of the macromolecule based on the peak time of the sample in the instrument. The green region represents the effective region, and the lines that do not appear within this region indicate impurities or interferences.

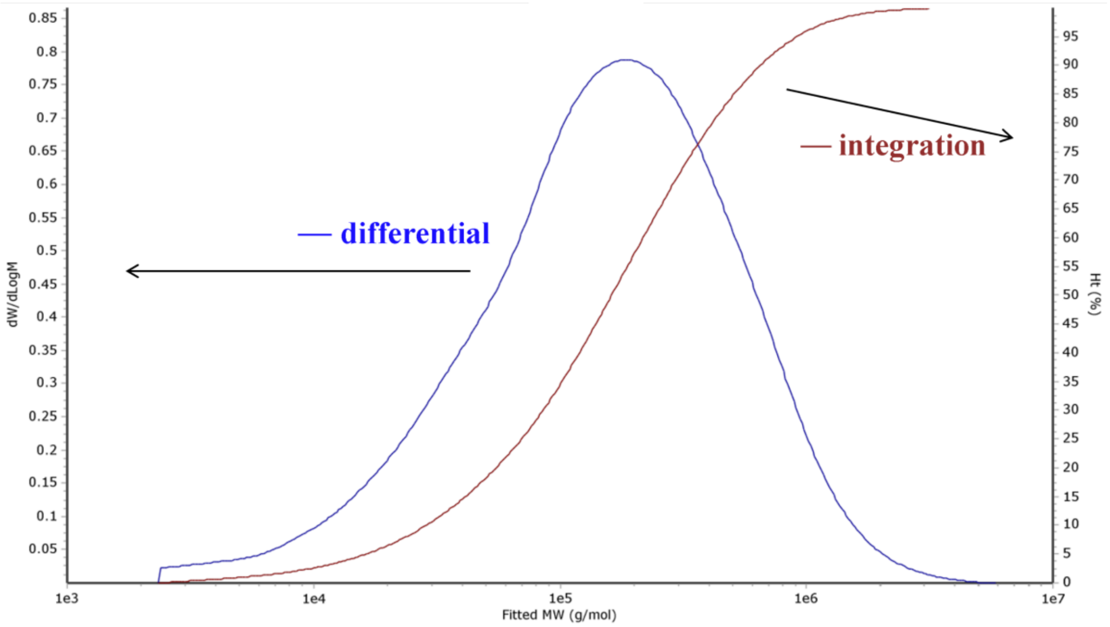

Figure S3. Molecular weight distribution diagram of polymer

In Figure S3, the x-axis represents the molecular weight, with the blue line depicting the proportion of the different molecular weights in the test sample, with the corresponding coordinates on the left side. The y-axis displays the proportion of these macromolecules. It is evident that the proportion of macromolecules in the sample with molecular weights within the range of 2.5e5 (Mw = $2.5*10^5$ g/mol) is the highest. The red line represents the cumulative distribution, with the corresponding coordinates on the right side. Let the horizontal axis of the plot be labeled as $1*10^6$ g/mol, with the vertical axis showing the proportion of macromolecules in the sample that fall within the molecular weight range of 0 g/mol to $1*10^6$ g/mol. It is evident that as the horizontal axis moves further to the right, the variety of macromolecular species increases, and their proportion approaches 100%.

Reference:

[1] Hao Chen, Gangqiang Zhang. Study on one-step synthesis of methyl

methacrylate [J]. Contemporary Chemical Industry, 2019, No.285(10):2306-2309.

[2] Wang W, Yan D . Reverse Atom Transfer Radical Polymerization Using AIBN or BPO as Initiator[J]. ACS Symposium Series, 2000, 768(s 2-3):263-275.

[3] Zhang L, Torkelson J M. Enhanced glass transition temperature of low molecular weight poly(methyl methacrylate) by initiator fragments located at chain ends[J]. Polymer, 2017, 122:194-199.

[4] Xia J, Matyjaszewski K. Controlled/"Living" Radical Polymerization. Homogeneous Reverse Atom Transfer Radical Polymerization Using AIBN as the Initiator[J]. Macromolecules, 1997, 30(25):7901-7910.

[5] Moineau G, Dubois P, R JérMe, et al. Alternative atom transfer radical polymerization for MMA using FeCl3 and AIBN in the presence of triphenylphosphine: an easy way to well-controlled PMMA[J]. Macromolecules, 1998, 31(2):545-547.

[6] Xianji Liu, Wenguang Ding, Ning Shi,etal. Synthesis of hydroxyl acrylic resin for powder coatings [J]. Shanghai Coatings, 2000, (02):7-10.

[7] Chuanxiang Qin, Jing Yang. Research and development prospect of acrylic powder coatings [J]. New Chemical Materials, 2004, (06):1-5.

[8] H Münstedt. Dependence of the Elongational Behavior of Polystyrene Melts on Molecular Weight and Molecular Weight Distribution[J]. Journal of Rheology, 1980, 24(6):847-867.

[9] Mccormick H W . Molecular weight distribution of polystyrene by sedimentation

velocity analysis[J]. Journal of Polymer Science, 1959, 36(130):341-349.
